# Receptor identification and in vivo efficacy of a lytic phage vB_EcoStr-FJ63A against colistin-resistant *Escherichia coli*

**DOI:** 10.1186/s13567-025-01687-6

**Published:** 2026-01-03

**Authors:** Tianshi Xiao, Cuncai Wang, Xiaolin Zhu, Xue Wang, Yimeng Fan, Xuchen Jia, Jianyu Lv, Tingting Chen, Zhihui Hao

**Affiliations:** State Key Laboratory of Veterinary Public Health and Safety, College of Veterinary Medicine, China Agricultural University, No. 2 Yuanmingyuan West Road, Beijing, 100193 China

**Keywords:** Bacteriophage, *E. coli*, adsorption, phage therapy

## Abstract

Bacteriophages (phages), as natural predators of bacteria, represent promising alternatives to antibiotics. However, challenges such as narrow host range and insufficient pharmacokinetic and pharmacodynamic (PK/PD) studies restrict phage therapy. In previous work, we isolated *Escherichia* phage vB_EcoStr-FJ63A, which exhibits a lytic activity against colistin-resistant *Escherichia coli* and obvious antibacterial activity in vitro. To elucidate its infection mechanism and evaluate its therapeutic potential, this study focused on identifying its host receptors and assessing its in vivo efficacy. By screening resistant mutants and analyzing the function of mutant sites, we hypothesized that outer membrane proteins (OMPs), lipopolysaccharide (LPS), or extracellular polysaccharides likely serve as receptor candidates for phage vB_EcoStr-FJ63A. The role of LPS as the receptor was supported by competitive binding and receptor removal assays, which demonstrated its necessity for phage adsorption. Bioinformatics analysis results provide evidences for long tail fiber adhesin of phage vB_EcoStr-FJ63A reversibly binds to OmpC, while its short tail fibers irreversibly anchor to LPS. Subsequent PK experiment in healthy mice revealed that a single intraperitoneal injection of phage vB_EcoStr-FJ63A (5 × 10^8^ PFU/mL or 5 × 10^6^ PFU/mL) achieved peak blood titers at 2 h post-administration, with phage remaining detectable for over 24 h (*n* = 6 per group at each time point). In bacteremic mice infected with colistin-resistant *E. coli*, both phage doses significantly reduced bacterial loads in the blood within 8 h (*n* = 3 per group at each time point) and improved survival rates (*n* = 6 per group) compared with the untreated control group. Collectively, our results provide evidence for the molecular basis of phage–host interactions for vB_EcoStr-FJ63A and provides data to advance phage therapy against antimicrobial resistant *E. coli*.

**Supplementary Information:**

The online version contains supplementary material available at 10.1186/s13567-025-01687-6.

## Introduction

*Escherichia coli* is an important opportunistic pathogen that affects both animal and human health. In livestock, pathogenic strains of *E. coli* can cause severe diseases, such as calf and piglet diarrhea, mastitis in dairy cows, and avian colibacillosis. These conditions result in significant economic losses due to increased mortality, reduced productivity, and increased treatment costs. The alarming rise of antimicrobial resistance (AMR) in animal-derived *E. coli* has further exacerbated this issue [1]. Colistin was once considered the last line of defense against multidrug-resistant Gram-negative bacteria, but the discovery of the plasmid-mediated colistin resistance gene *mcr-1* has raised concerns about the advent of a post-antibiotic era [2]. Therefore, there is an urgent need to find new therapeutic approaches to resolve this problem.

Bacteriophages (phages), as natural predators of bacteria, are viruses that can specifically infect and lyse bacteria. Compared with traditional antibiotics, phages can self-replicate and have minimal impact on human and animal hosts, making them a promising alternative or complementary treatment to conventional antibiotics. Several recent studies and compassionate use cases have demonstrated the potential efficacy of phage therapy, and reported adverse events are generally mild and rare; however, immune responses and variable treatment outcomes have been observed in some clinical contexts [3–5]. However, phage therapy has not yet gained widespread application owing to certain inherent limitations. The most significant limitation is the high host specificity of phages. Phages typically only infect a few strains of a particular bacterial species, which severely restricts their widespread use and makes it difficult to combat infections caused by multiple bacterial species.

Another major scientific barrier is the lack of understanding of the pharmacokinetics (PK) and pharmacodynamics (PD) of bacteriophages in vivo [6]. Antibiotics have long served as the “reference standard” for treating bacterial infections, with their pharmacokinetic and pharmacodynamic (PK/PD) extensively characterized [7]. In contrast, the PK/PD of phage therapy is not well-established and is significantly complicated by phage self-replication, adsorption rates, and clearance by host immunity. Furthermore, variations in phage types, preparations, and treatment regimens hinder drawing universal conclusions about the in vivo PK/PD characteristics of phages [8].

In our previous work, we isolated the *Escherichia* phage vB_EcoStr-FJ63A from pig fecal samples, a new member of the genus *Krischvirus* in the family *Straboviridae* [9]. This phage exhibited a broad lytic spectrum and antibacterial activity against colistin-resistant *E. coli* in vitro. Therefore, the aim of this study was to identify and validate the host receptor of phage vB_EcoStr-FJ63A by investigating resistance mutations of the host and to evaluate the antibacterial efficacy and pharmacokinetics of phage vB_EcoStr-FJ63A in mice, contributing to the development of effective phage therapy for infections caused by AMR bacteria.

## Materials and methods

### Bacteria strains and experimental animals

The *E. coli* BL21(DE3) strain was provided by Beijing Tsingke Biotechnology Co., Ltd (Beijing, China). The colistin-resistant *E. coli* strains 63 and RN24 were respectively isolated from chicken samples in Shandong Province and dog samples in Guangxi Province, China. All bacterial strains (Additional file 1) used in this study were stored in 40% (*v*/*v*) glycerol at −80 ℃. For propagation, all strains were cultivated in Luria–Bertani (LB) broth at 37 ℃ with shaking at 180 rpm overnight. The animal experiment design was based on previous studies by Dhungana et al., Chow et al., and Lin et al. [6, 10, 11]. A total of 169 female BALB/c mice (specific pathogen-free [SPF] grade, 6–8 weeks old, 14–17 g) were purchased from Beijing Beiyou Biotechnology Co., Ltd (Beijing, China). All mice were group-housed (six mice per cage) in individually ventilated cages within a SPF facility under a strict 12-h light/dark cycle at a temperature of 25 ± 2 ℃. They had free access to a standard commercial diet and sterile water. The assignment of mice to experimental groups was randomized for all in vivo studies. Owing to the practical constraints of the study setup and the observable differences between the treatment (phage suspension) and control (phosphate-buffered saline [PBS] buffer) solutions, the experiments were not conducted in a blinded manner. All animal experiments were conducted in strict accordance with the requirements of the Laboratory Animal Welfare and Animal Experiment Ethics Committee of China Agricultural University (approval number AW71112202-2-1).

### Cultivation and purification of phage vB_EcoStr-FJ63A

The host strain *E. coli* 63 was cultured overnight in LB broth at 37 ℃. Then, 4 mL of overnight culture was added to 36 mL of fresh LB broth and incubated at 37 ℃ with shaking at 180 rpm for 1 h. Then, 100 μL of high-titer phage vB_EcoStr-FJ63A (> 10^8^ PFU/mL) was added, and the mixture was incubated for approximately 5 h at 37 ℃ with shaking at 180 rpm. The lysate was centrifuged twice at 8000 × *g* for 30 min at 4 ℃. The supernatant was filtered through a 0.22 μm sterile membrane, mixed with 4 mL chloroform, vortexed thoroughly, incubated at room temperature for 10 min, and centrifuged at 4000 × *g* for 5 min [12]. The upper aqueous phase was collected and stored at 4 ℃.

For purification, 8 mL of 1-octanol was added to the 20 mL phage suspension, shaken at room temperature for 1 h, and incubated at 4 ℃ for 1.5 h [13]. After centrifugation at 4000 × *g* for 10 min, the bottom aqueous phase was collected. The phage suspension was then concentrated and exchanged into SM buffer using a VivaFlow 200 cross-flow filtration (CFF) system [14]. The system was pre-rinsed with 500 mL ultrapure water, and a total of approximately 480 mL phage suspension was circulated until the volume was reduced to approximately 200 mL. SM buffer (~2.5 L total) was continuously added until the solution became clear and colorless, followed by concentration to the desired volume.

The concentrated phage suspension was passed through Pierce^™^ High Capacity Endotoxin Removal Spin Columns three times to further remove LPS [14]. The final purified phage concentrate was filtered through a 0.22 μm sterile membrane, and endotoxin levels were quantified using the Pierce^™^ Chromogenic Endotoxin Quant Kit. The endotoxin level in the final preparation was approximately 1.2 × 10^4^ EU/mL.

### Screening of phage-resistant mutant strains

On the basis of the previously established time–kill curve, phage vB_EcoStr-FJ63A and the host bacteria were mixed at different multiplicities of infection (MOI) and incubated at 37 ℃ with shaking at 180 rpm for 2 h until reaching the time point of maximal bactericidal activity [9]. Then, a 100 μL sample was taken and spread onto the surface of LB agar plates, followed by incubation at 37 ℃ overnight. Single colonies were picked from the LB agar plates and subcultured for three consecutive generations. The susceptibility of these strains to the phage was assessed using the double-layer agar plate method. To evaluate the adsorption efficiency of phage vB_EcoStr-FJ63A to the resistant mutant strains, 100 μL of phage suspension was mixed with 100 μL of the resistant mutant strains in 800 μL of LB broth and incubated at 37 ℃ for 15 min. The mixture was centrifuged at 14 000 × *g* for 1 min at 4 ℃. The supernatant was transferred to a new centrifuge tube, and the pellet was resuspended in 1 mL of PBS buffer. Phage titers in both the supernatant and resuspended pellet were determined.

### Sequencing and analysis of resistant mutant strains

The host strain *E. coli* 63 and the resistant mutant strains were cultured at 37 ℃ with shaking at 180 rpm until reaching the logarithmic growth phase. The cultures were centrifuged at 4000 × *g* for 10 min at 4 ℃, and the supernatant was discarded. The bacterial pellets were washed twice with ultrapure water, flash-frozen on dry ice, and sent to Beijing Sinobiocore Biotechnology Co., Ltd for sequencing using Illumina NovaSeq 6000 and Oxford Nanopore PromethION platforms. The sequencing data were assembled using Unicycler [15]. The assembled host genomes was functionally annotated using the RAST server [16]. The genbank file of *E. coli* 63 was used as the reference genome for subsequent analysis. Mutations in the resistant strains were identified by aligning their sequencing data to the reference genome using breseq [17] and Snippy [18].

### Analysis of receptor types of vB_EcoStr-FJ63A

The host strain *E. coli* 63 was cultured to the logarithmic growth phase and centrifuged at 8000 × *g* for 1 min at 4 ℃. The bacterial pellet was washed twice with PBS buffer and resuspended in an equal volume of PBS buffer. To degrade bacterial surface proteins, 0.1 volume of 1 mg/mL protease K was added to the cell suspension and incubated at 37 ℃ for 3 h [19]. To oxidize surface polysaccharides, 0.1 volume of 100 mM sodium periodate was added to the cell suspension and incubated in the dark at room temperature for 2 h [20]. For combined treatment, both protease K and sodium periodate were sequentially applied. After treatment, the cells were centrifuged at 8000 × *g* for 1 min at 4 ℃, and the bacterial pellet was washed twice with PBS buffer. Then, 100 μL of phage vB_EcoStr-FJ63A (10^6^ PFU/mL) was respectively mixed with 100 μL of protease-K-treated, sodium-periodate-treated, or co-treated bacterial suspension in 800 μL LB broth and incubated at 37 ℃ with shaking at 180 rpm for 15 min. The mixture was centrifuged at 14 000 × *g* for 1 min at 4 ℃. The supernatant was transferred to a new centrifuge tube, and the pellet was resuspended in 1 mL PBS buffer. Phage titers in the supernatant and resuspended pellet were determined using the double-layer agar plate method.

### Effect of LPS on the adsorption of phage vB_EcoStr-FJ63A to the host

LPS was extracted from the host strain *E. coli* 63 and the resistant mutant strains using the Solarbio LPS Extraction Kit. Phage vB_EcoStr-FJ63A was mixed with 0.1 volume of extracted LPS and incubated at 37 ℃ for 15 min, respectively. The control group was prepared by mixing the phage with PBS buffer at the same volume. Then, 100 μL of each mixture was combined with 100 μL of the overnight culture of the host bacteria in 800 μL of LB broth and incubated at 37 ℃ for 15 min. The samples were then centrifuged at 14 000 × *g* at 4 ℃ for 1 min. The supernatant was transferred to a new centrifuge tube, and the pellet was resuspended in 1 mL of PBS buffer. Phage titers in both the supernatant and the resuspended pellet were determined.

### Expression and purification of OmpC and OmpC-Gln172*

The prokaryotic expression and purification of OmpC and OmpC-Gln172* were performed by Beijing Tsingke Biotech Co., Ltd (Beijing, China). The nucleic acid sequences coding for the full-length *ompC* of *E. coli* 63 and the truncated *ompC*-Gln172* of *E. coli* 63M100R1, which were codon-optimized and cloned into the pET-28a plasmid (Additional file 2). The plasmid pET-28a was used as the expression vector, and *E. coli* BL21 (DE3) served as the expression host.

For transformation, 1 μL of plasmid was mixed with 100 μL of competent cells, incubated on ice for 20 min, subjected to heat shock at 42 ℃ for 90 s, and then immediately placed on ice for 5 min. After adding 600 μL of LB broth, the mixture was incubated at 37 ℃ with shaking at 180 rpm for 1 h. The transformed cells were then plated onto LB agar containing 50 μg/mL kanamycin and incubated at 37 ℃ overnight.

A single colony was inoculated into 4 mL of LB broth with 50 μg/mL kanamycin and cultured overnight at 37 ℃. This culture was diluted 1:100 into 100 mL of fresh LB medium with kanamycin and incubated until the OD_600_ reached 0.5–0.8. Protein expression was induced by adding 0.2 mM IPTG, followed by incubation at 16 ℃ with shaking at 180 rpm for 18 h.

The cells were harvested by centrifugation at 4000 × *g* for 10 min. The pellet was resuspended in PBS buffer, and phenylmethylsulfonyl fluoride (PMSF) was added to a final concentration of 1 mM. The cells were lysed by sonication, and the supernatant and pellet fractions were analyzed by 12% SDS-PAGE.

For purification, the protein solution was applied at 1 mL/min to a Ni–NTA affinity column pre-equilibrated with Binding Buffer (10 mM PBS, 0 mM imidazole, 8 M urea, pH 7.4). The column was washed with Binding Buffer until the OD_280_ reached baseline. The target protein was eluted using a stepwise imidazole gradient (25, 50, 100, and 200 mM in 10 mM PBS, 8 M urea, pH 7.4) at a flow rate of 1 mL/min. The eluted protein was refolded by gradient dialysis in PBS buffers with decreasing urea concentrations (6 M, 4 M, 2 M, and 0 M).

### Effect of OmpC on the adsorption of phage vB_EcoStr-FJ63A to the host

Phage vB_EcoStr-FJ63A was mixed with either purified OmpC or OmpC-Gln172* at 1:1 (*v*:*v*) and incubated at 37 ℃ for 15 min. The control group was prepared by mixing the phage with PBS buffer under the same conditions. Subsequently, 100 μL of each mixture was combined with 100 μL of 100 mM sodium-periodate-treated host strain in 800 μL LB broth and incubated at 37 ℃ for 15 min. The mixture was centrifuged at 14 000 × *g* for 1 min at 4 ℃. The supernatant was transferred to a new centrifuge tube, and the pellet was resuspended in 1 mL PBS buffer. Phage titers in both the supernatant and resuspended pellet were determined.

### Protein structure prediction and protein–protein interaction modeling

Homologous structural information for the long tail fiber adhesin and the OmpC were retrieved from SWISS-MODEL [21] and the AlphaFold Protein Structure Database [22] (Additional file 3). On the basis of this information, the protein structures were predicted using AlphaFold Server, and the interaction between the long tail fiber adhesin and OmpC was simulated [23]. The results were analyzed and visualized using ChimeraX [24].

### Pharmacokinetics of phage vB_EcoStr-FJ63A in mice blood

A total of 84 female BALB/c mice were randomly divided into high-dose and low-dose groups, with 42 mice in each group [10]. Mice in the high-dose treatment (HT) group and low-dose treatment (LT) group were intraperitoneally injected with 200 μL of phage vB_EcoStr-FJ63A at concentrations of 2.5 × 10^9^ PFU/mL and 2.5 × 10^7^ PFU/mL, respectively. At 30 min, 1 h, 2 h, 4 h, 8 h, 24 h, and 48 h post-injection, six mice from each group were anesthetized with Zoletil 50, and venous blood was collected via the orbital sinus under aseptic conditions. All blood samples were serially diluted tenfold with PBS buffer, spotted onto double-layer agar plates, and incubated at 37 ℃ overnight for plaque counts. PK parameters were estimated by non-compartmental analysis using Phoenix (version 8.3, Certara, Inc.).

### Establishment of an *E. coli* infection model

The phage-sensitive strain *E. coli* RN24 was used to establish an *E. coli* infection model owing to its pathogenicity (Additional file 4). The *E. coli* RN24 was inoculated into LB broth and incubated at 37 ℃ with shaking at 180 rpm overnight. Then, 18 mL of fresh LB broth was mixed with 2 mL of the overnight *E. coli* RN24 culture and incubated at 37 ℃ with shaking at 180 rpm for 6 h until reaching the mid-logarithmic growth phase. The culture was centrifuged at 8000 × *g* for 1 min at 4 ℃, and the pellet was washed twice with PBS buffer. The pellet was resuspended in PBS buffer and serially diluted with PBS buffer. A total of 16 female BALB/c mice were randomly divided into four groups (4 mice per group). Each group received an intraperitoneal injection of 100 μL *E. coli* RN24 at concentrations of 10^10^ CFU/mL, 10^9^ CFU/mL, 10^8^ CFU/mL, or 10^7^ CFU/mL, respectively. Mice were monitored for 72 h. The absolute lethal dose (LD_100_) was defined as the lowest bacterial concentration resulting in 100% mortality, while the minimum lethal dose (MLD) was defined as the lowest concentration causing any mortality.

### Evaluation of therapeutic efficacy of phage vB_EcoStr-FJ63A

A total of 24 female BALB/c mice were randomly divided into four groups (6 mice per group): *E. coli* infection model group, high-dose treatment group, low-dose treatment group, and blank control group. Mice in the infection model group, high-dose group, and low-dose group were intraperitoneally injected with 100 μL of *E. coli* RN24 at the LD_100_. The blank control group received an equivalent volume of PBS buffer. Two hours post-infection, the high-dose and low-dose treatment groups were injected intraperitoneally with 200 μL of phage vB_EcoStr-FJ63A at 2.5 × 10^9^ PFU/mL and 2.5 × 10^7^ PFU/mL, respectively, at a site contralateral to the bacterial challenge. The infection model and blank control groups received 200 μL PBS buffer. The clinical symptoms, body weight changes, and survival rates were monitored over 72 h.

### Antimicrobial activity and titer dynamics of phage vB_EcoStr-FJ63A in mice blood

A total of 45 female BALB/c mice were randomly divided into three groups (15 mice per group): high-dose treatment group, low-dose treatment group, and *E. coli* infection model group. All mice were intraperitoneally injected with 100 μL of *E. coli* RN24 at the MLD. Two hours post-infection, the high-dose and low-dose groups received 200 μL of phage vB_EcoStr-FJ63A at 2.5 × 10^9^ PFU/mL and 2.5 × 10^7^ PFU/mL, respectively, at a site contralateral to the bacterial challenge. At the same time, the infection model group received 200 μL PBS buffer. At 0 h, 1 h, 2 h, 4 h, 8 h, and 24 h post-phage injection, three mice from each group were anesthetized with Zoletil 50, and venous blood was collected via the orbital sinus under aseptic conditions. All blood samples were serially diluted tenfold with PBS buffer, and spotted onto LB agar plates and double-layer agar plates to determine the bacterial concentration and phage titer.

### Statistical analysis

The experimental data in this study were statistically analyzed using GraphPad Prism (version 10.1.2, GraphPad Software, Inc.). Data were presented as means ± standard deviation (SD). The results of the adsorption assay were analyzed using one-way analysis of variance (ANOVA). For comparisons of continuous data across multiple groups (e.g., phage titers, bacterial loads), two-way ANOVA was used. Survival data were analyzed using the Kaplan–Meier method, and statistical differences between groups were assessed with the log-rank test. The significance level was set at *p* < 0.05. *p* > 0.05 (ns), 0.01 < *p* < 0.05 (*), 0.001 < *p* < 0.01 (**), 0.0001 < *p* < 0.001 (***), and *p* < 0.0001 (****).

## Results

### Screening of phage-resistant mutant strains

**Figure 1:**
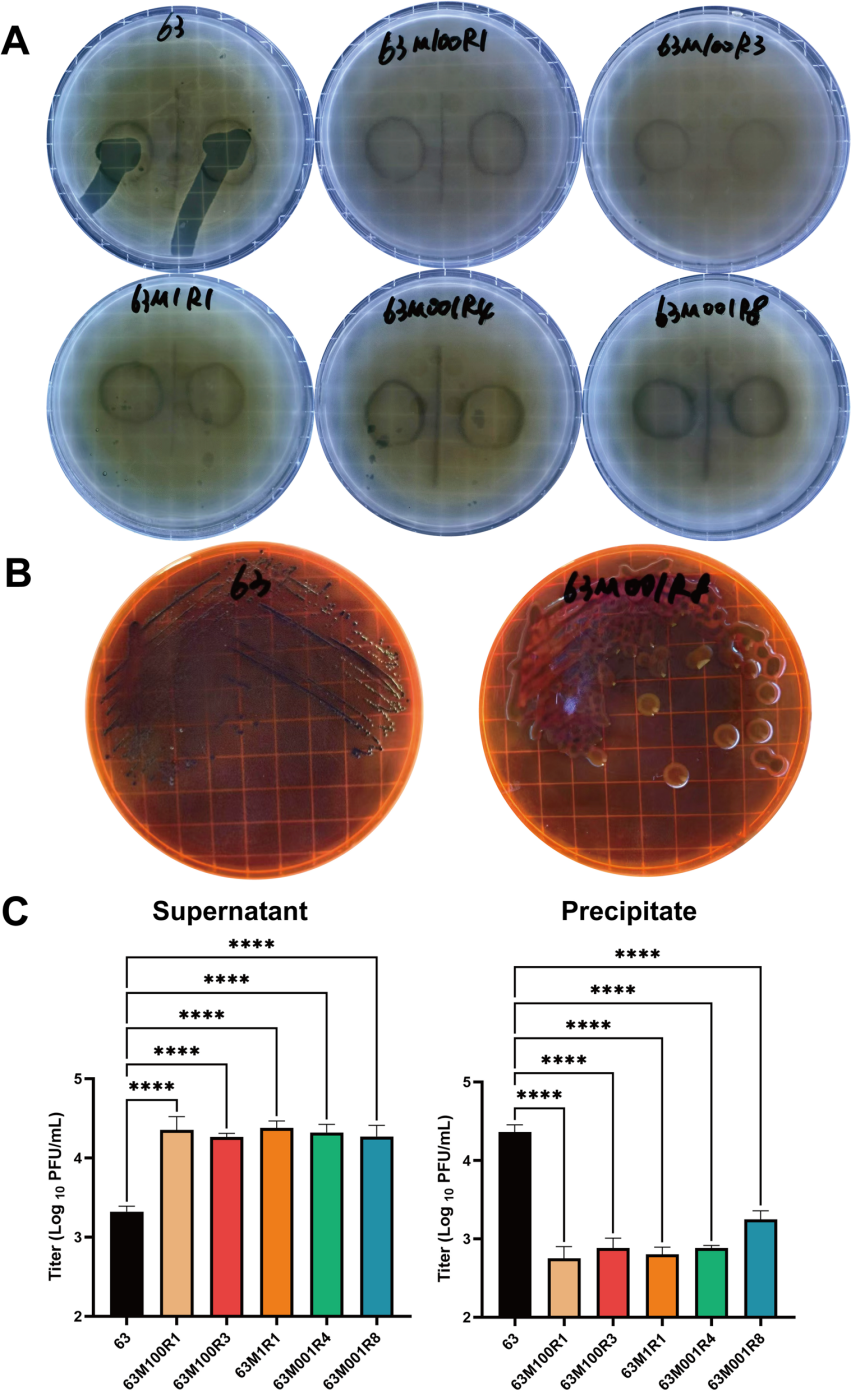
**Screening and identification of resistant mutant strains**. **A** Phage vB_EcoStr-FJ63A failed to lyse resistant mutant strains to form plaques. **B** The resistant mutant strain 63M001R8 exhibited a mucoid phenotype. **C** Phage vB_EcoStr-FJ63A showed reduced adsorption capacity toward the resistant mutant strains. Data are presented as means ± SD (*n* = 3 biological replicates). Statistical significance was determined by one-way ANOVA followed by Dunnett’s multiple comparisons test (versus *E. coli* 63). Significance levels: ****p < 0.0001.

After mixing phage vB_EcoStr-FJ63A with *E. coli* 63 at different MOI and incubated at 37 ℃ for 2 h, samples were plated on LB agar plates. Following overnight incubation, individual bacterial colonies growing on the plates were selected, purified, and tested for phage susceptibility. Results showed that phage vB_EcoStr-FJ63A could not form plaques on LB agar plates inoculated with strains 63M100R1, 63M100R3, 63M1R1, 63M001R4, or 63M001R8 (Figure 1A). The colony morphologies of 63M100R1, 63M100R3, 63M1R1, and 63M001R4 resembled that of *E. coli* 63, whereas 63M001R8 exhibited a mucoid phenotype (Figure 1B).

Phage adsorption assays for the resistant mutant strains are shown in Figure 1C. After mixing phage vB_EcoStr-FJ63A with the five resistant mutant strains and incubating at 37 ℃ for 15 min, 91.6% of phages adsorbed to the surface of the host *E. coli* 63. In contrast, over 90% of phages remained free in the culture medium, failing to adsorb to the surfaces of the five resistant mutant strains. The phage titers in the supernatants of the resistant mutant strain groups were significantly higher than those in the *E. coli* 63 control group, with *p* values of 0.0062, 0.0283, 0.0039, 0.0111, and 0.0251, respectively. These results indicate that the phage resistance of the mutant strains primarily arises from their ability to inhibit phage vB_EcoStr-FJ63A adsorption.

### Genomic analysis of phage-resistant mutant strains

Mutations in the resistant mutant strains were analyzed using breseq and Snippy (Table 1). In strain 63M100R1, nucleotide 514 of *ompC* changed from C to T, resulting in the conversion of Gln at position 172 to a stop codon. In *etk*, nucleotide 1102 changed from A to C, leading to the substitution of Thr at position 368 with Pro. For strain 63M100R3, nucleotide 658 of *ompR* changed from C to T, causing Arg at position 220 to be replaced by Cys. In strain 63M1R1, a deletion of nucleotides 138–151 in *etk* induced a frameshift mutation at amino acid position 47. In addition, two mutations were identified in *insB*: one synonymous mutation and another that converted amino acid 98 to a stop codon. In strain 63M001R4, the deletion of nucleotide 62 (T) in *wyz* caused a frameshift mutation at Leu position 21, while nucleotide 929 of *ugd* changed from C to G, resulting in Ser at position 310 being replaced by a stop codon. For strain 63M001R8, nucleotide 1558 of *yrfF* changed from T to A, altering Trp at position 520 to Arg, and nucleotide 587 of *yfcC* changed from A to C, substituting Asn at position 196 with Thr.

**Table 1:** **Mutation site analysis of resistant mutant strains and their gene function annotation**

Strain	Contig	Position	Gene	Alternate	Effect	Product
63M100R1	1	1648455	*ompC*	514C > T	Stop gained Gln172*	Outer membrane porin OmpC
	1	3009237	*etk/yccC*	1102A > C	Missense variant Thr368Pro	Tyrosine-protein kinase etk
63M100R3	1	351766	*ompR*	658C > T	Missense variant Arg220Cys	Two-component system response regulator OmpR
63M1R1	1	3008265	*etk/yccC*	138_151delCGCTTACTCGCTGT	Frameshift variant Ala47fs	Tyrosine-protein kinase etk
	1	4122194	*insB*	252G > A	Synonymous variant Glu84Glu	IS1 protein InsB
	1	4122153	*insB*	293G > A	Stop gained Trp98*	IS1 protein InsB
63M001R4	1	1845482	*wyz*	62delT	Frameshift variant Leu21fs	O8 family O-antigen polymerase
	1	1851751	*ugd*	929C > G	Stop gained Ser310*	UDP-glucose 6-dehydrogenase
63M001R8	1	359667	*yrfF*	1558 T > A	Missense variant Trp520Arg	Membrane protein IgaA homolog
	1	1547390	*yfcC*	587A > C	Missense variant Asn196Thr	putative basic amino acid antiporter YfcC

*fs* frameshift, frameshift mutations; * termination codon, nonsense mutation.

Among the mutated genes, the outer membrane protein OmpC may, as annotated in the UniProt database, serves as a putative receptor mediating phage attachment to host cells, and OmpR regulates osmotic adaptation and controls *ompC* transcription. Tyrosine-protein kinase Etk is involved in extracellular polysaccharide biosynthesis and biofilm formation. O-antigen polymerase participates in O-antigen polysaccharide synthesis. UDP-glucose 6-dehydrogenase (UgD) contributes to the biosynthesis of colanic acid, LPS, and UDP-glucuronic acid. The putative membrane protein IgaA homolog regulates the phosphorelay signaling system and influences extracellular polysaccharide synthesis and secretion. These findings suggest that the adsorption of phage vB_EcoStr-FJ63A to *E. coli* 63 may involve interactions with outer membrane proteins, LPS, and extracellular polysaccharides.

### Determine the receptor types of phage vB_EcoStr-FJ63A

**Figure 2:**
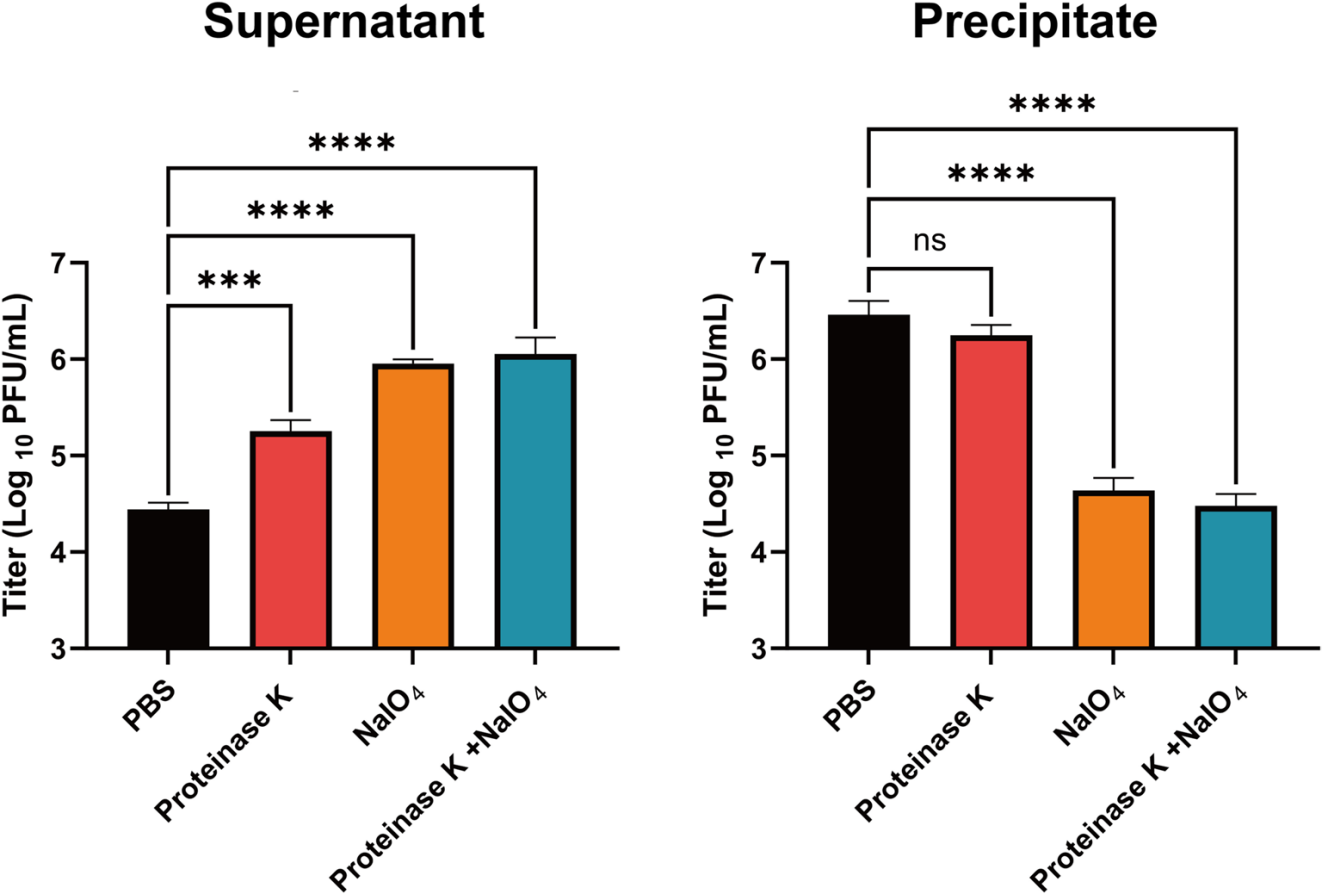
**Analysis of the receptor type for host adsorption by phage vB_EcoStr-FJ63A**. Data are presented as means ± SD (*n* = 3 biological replicates). Statistical significance was determined by one-way ANOVA followed by Dunnett’s multiple comparisons test (versus PBS). Significance levels: ***p < 0.001; ****p < 0.0001; ns, not significant.

The adsorption of phage vB_EcoStr-FJ63A to *E. coli* 63 after disrupting its outer membrane proteins and surface polysaccharides with proteinase K and sodium periodate, respectively, is shown in Figure 2. Although the phage titer in the supernatant of the proteinase-K-treated group was higher than that of the PBS-treated control group, the difference was not statistically significant (*p* = 0.8461). In contrast, the phage titers in the supernatants of the sodium-periodate-treated group and the sodium periodate + proteinase-K-treated group were significantly higher than those of the PBS-treated control group, with *p* values of 0.0132 and 0.0034, respectively. Results revealed that approximately 99.1% of phages adsorbed to the PBS-treated control, 90.8% adsorbed to the host with disrupted outer membrane proteins, 4.6% adsorbed to the host with disrupted surface polysaccharides, and only 2.6% adsorbed to the host with both outer membrane proteins and surface polysaccharides disrupted. These results indicate that both outer membrane proteins and surface polysaccharides serve as putative receptors for phage vB_EcoStr-FJ63A, but surface polysaccharides play a predominant role in phage adsorption.

### Effect of LPS on the adsorption of phage vB_EcoStr-FJ63A to the host

**Figure 3:**
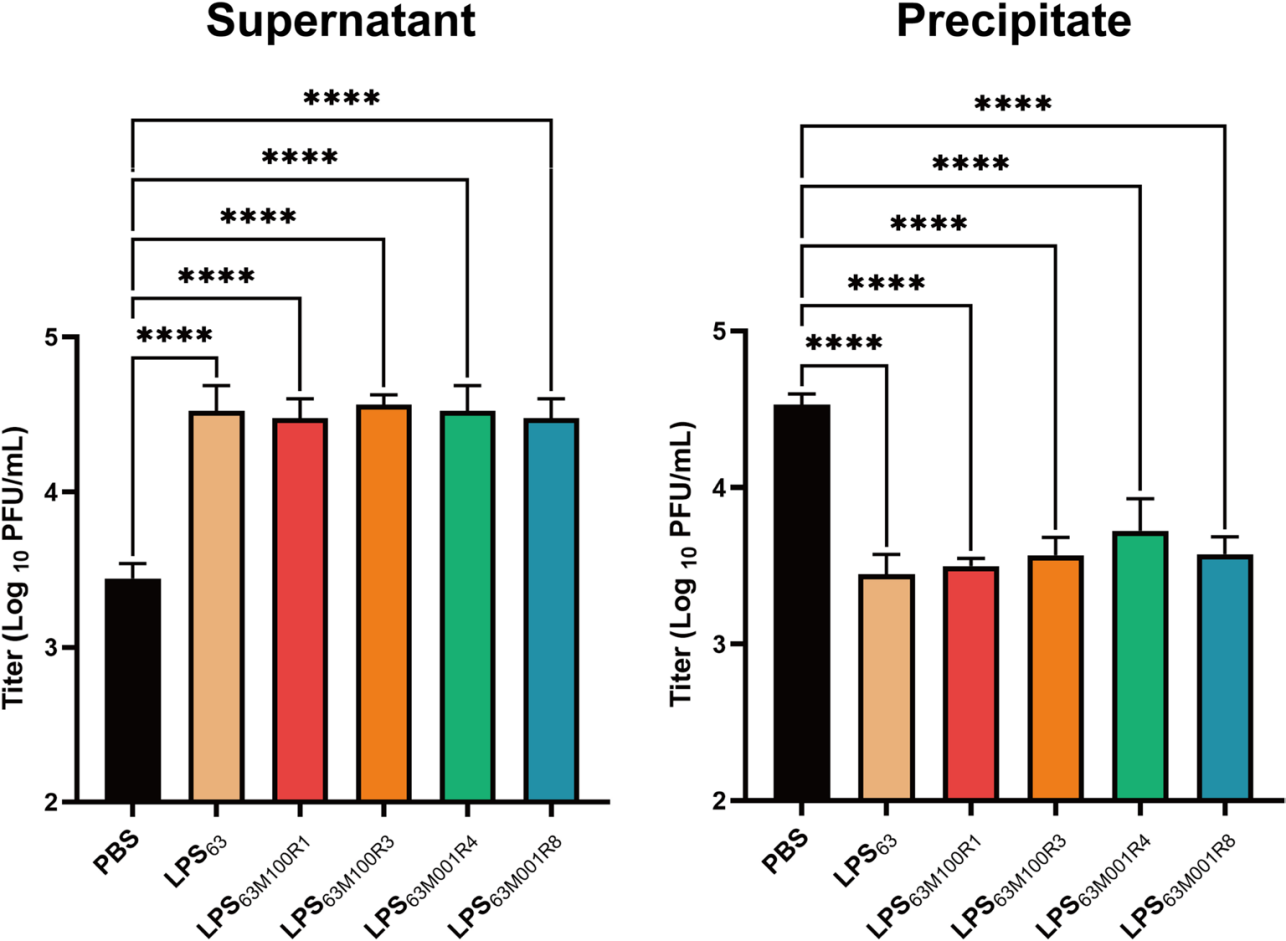
**Effect of exogenous LPS addition on the adsorption of phage vB_EcoStr-FJ63A to the host**. Data are presented as means ± SD (*n* = 3 biological replicates). Statistical significance was determined by one-way ANOVA followed by Dunnett’s multiple comparisons test (versus PBS). Significance levels: ****p < 0.0001.

Phage vB_EcoStr-FJ63A was mixed with LPS extracted from the host *E. coli* 63 and the five resistant mutant strains and incubated at 37 ℃ for 15 min. The adsorption of treated phages to the host is shown in Figure 3. LPS extracted from *E. coli* 63 and all five resistant mutant strains exhibited a competitive inhibitory effect on phage adsorption, preventing phages from binding to the host. The phage titers in the supernatants of all LPS-treated groups were significantly higher than those of the PBS-treated control group, with *p* values of 0.0184, 0.0360, 0.0095, 0.0184, and 0.0360, respectively. These findings confirm that LPS is one of the binding sites for phage vB_EcoStr-FJ63A during adsorption and that the resistant mutant strains did not affect phage adsorption by altering LPS.

### Effect of OmpC on the adsorption of phage vB_EcoStr-FJ63A to the host

**Figure 4:**
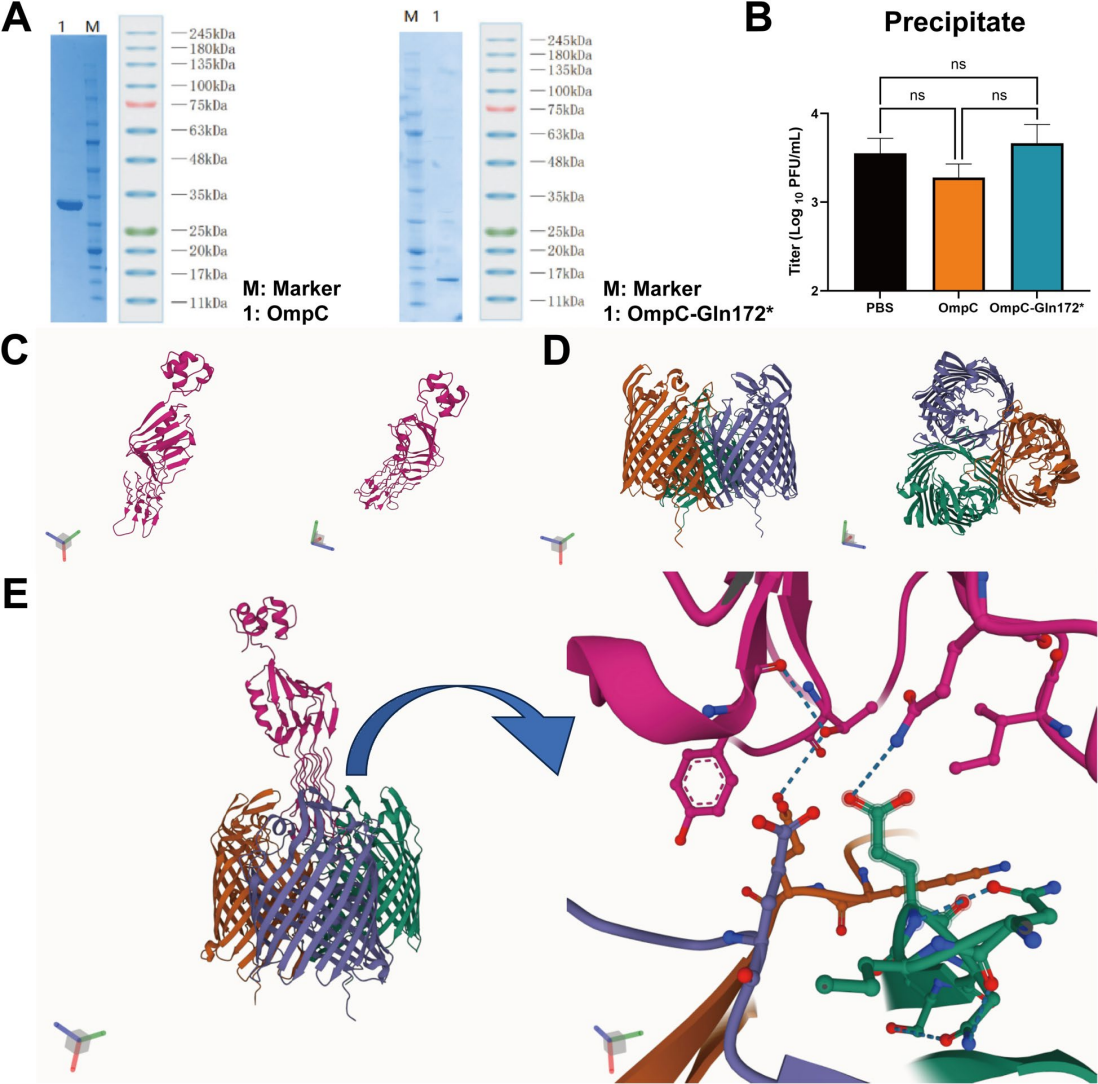
**OmpC may be the adsorption target of phage vB_EcoStr-FJ63A**. **A** Expression and purification of OmpC from *E. coli* 63 and OmpC-Gln172* from the resistant mutant strain 63M100R1. **B** Effect of exogenously added OmpC and OmpC-Gln172* on the adsorption of phage vB_EcoStr-FJ63A to the host. Data are presented as means ± SD (*n* = 3 biological replicates). Statistical significance was determined by one-way ANOVA followed by Dunnett’s multiple comparisons test. Significance levels: ns, not significant. **C** Predicted structure of the long tail fiber adhesin of phage vB_EcoStr-FJ63A. **D** Predicted structure of the OmpC trimer from *E. coli* 63. **E** AlphaFold3 predicted that the adhesin of phage vB_EcoStr-FJ63A inserts into the cavity formed by the OmpC trimer of *E. coli* 63 and forms hydrogen bonds.

The OmpC of the host strain *E. coli* 63 and the truncated OmpC-Gln172* from the resistant mutant strain 63M100R1 were successfully cloned into the plasmid pET28a and expressed in *E. coli* BL21(DE3). During expression, OmpC formed inclusion bodies, which were refolded using urea gradient dialysis. SDS-PAGE analysis confirmed the molecular weight of refolded OmpC as 40.8 kDa (Figure 4A). Purified OmpC-Gln172* was verified by SDS-PAGE with a molecular weight of 18.2 kDa (Figure 4A). To minimize interference from surface polysaccharides, *E. coli* 63 was pretreated with sodium periodate. Phage vB_EcoStr-FJ63A was mixed with OmpC or OmpC-Gln172* and incubated at 37 ℃ for 15 min. Adsorption of treated phages to sodium-periodate-pretreated *E. coli* 63 is shown in Figure 4B. The adsorption rates were 1.7% for OmpC-treated phages, 7.2% for OmpC-Gln172*-treated phages, and 5.3% for the PBS-treated control. Although OmpC treatment slightly reduced phage adsorption compared with the other groups, no statistically significant differences were observed. These results suggest that OmpC may serve as one of the potential binding sites for the phage.

Phage long tail fiber adhesin typically exist as monomers at the distal end of the tail fibers. AlphaFold3 modeling predicted the structure of the adhesin monomer (Figure 4C) with a predicted template modeling score (pTM) of 0.9. OmpC generally forms trimers, and AlphaFold3 modeling of the trimer (Figure 4D) yielded an interface pTM (ipTM) of 0.89 and a pTM of 0.91. A pTM score above 0.5 indicates that the overall predicted fold of the complex likely resembles the true structure. The ipTM metric evaluates the accuracy of inter-subunit positioning within the complex: scores above 0.8 represent high-confidence predictions, below 0.6 suggest potential prediction failures, and scores between 0.6 and 0.8 fall in a gray zone. Thus, the structural predictions for the OmpC trimer and long tail fiber adhesin are highly credible. Further modeling of the adhesin bound to the OmpC trimer (Figure 4E) yielded an ipTM of 0.58 and a pTM of 0.67, suggesting a potential interaction. ChimeraX analysis revealed 81 interactions between the OmpC trimer and the adhesin, including eight hydrogen bonds.

### Pharmacokinetics of phage vB_EcoStr-FJ63A in mice blood

**Figure 5:**
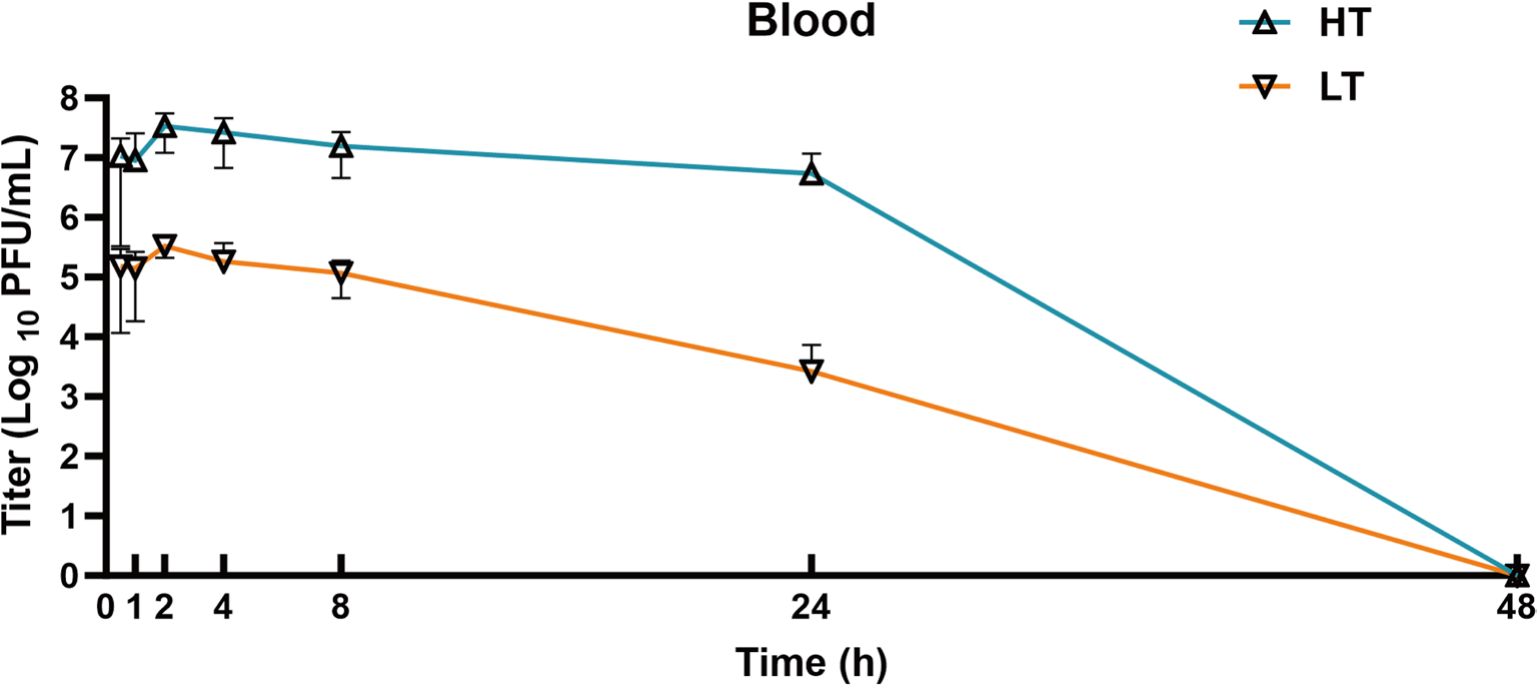
**The pharmacokinetics of phage vB_EcoStr-FJ63A in the blood of healthy mice (*n* = 6).**

The endotoxin content in the phage vB_EcoStr-FJ63A solution was reduced to 1.2 × 10^4^ EU/mL through 1-octanol extraction, tangential flow filtration, and LPS affinity chromatography. The phage titer decreased from 10.18 log _10_ PFU/mL to 9.40 log _10_ PFU/mL, with a recovery rate of 16.67%. As shown in Figure 5, phage vB_EcoStr-FJ63A was detected in blood of mice 30 min after intraperitoneal injection of high-dose (5 × 10^8^ PFU) or low-dose (5 × 10^6^ PFU) treatments. Both dose groups showed similar trends: phage titers peaked at 2 h post-injection, reaching 7.53 log _10_ PFU/mL (high-dose) and 5.52 log _10_ PFU/mL (low-dose). The titers subsequently declined, with the high-dose group retaining relatively high levels (a decrease of ~0.79 log _10_ PFU/mL) at 24 h, while the low-dose group exhibited a more pronounced reduction (2.09 log _10_ PFU/mL). By 48 h, no phage was detectable in the blood of either group. Pharmacokinetic analysis using Phoenix 8.3.5 revealed that the half-life (T_1/2_) of phage vB_EcoStr-FJ63A in healthy mice was approximately 0.69 h, independent of the administered dose, though the mean residence time (MRT), apparent volume of distribution (Vd), and clearance (CL) varied with dosage (Table 2).

**Table 2:** **Pharmacokinetic parameters of vB_EcoStr-FJ63A in healthy mice**

Parameters	Blood	
Dose (PFU/mL)	5 × 10^6^	5 × 10^8^
*T*_max_ (h)	2	2
*C*_max_ (PFU/mL)	3.33 × 10^5^	3.37 × 10^7^
λz	0.29	0.38
T_1/2_/λz	2.35	1.8
T_1/2_ (h)	0.69	0.69
AUC_0–t_ (PFU·h/mL)	2.46 × 10^6^	4.08 × 10^8^
AUC_inf_ (PFU × h/mL)	2.46 × 10^6^	4.08 × 10^8^
AUMC_0-t_ (PFU × h/mL)	1.40 × 10^7^	4.31 × 10^9^
MRT (h)	5.69	10.55
Vd (L)	0.0069	0.0032
CL	2.04	1.23

### Therapeutic efficacy of phage vB_EcoStr-FJ63A in vivo

**Figure 6:**
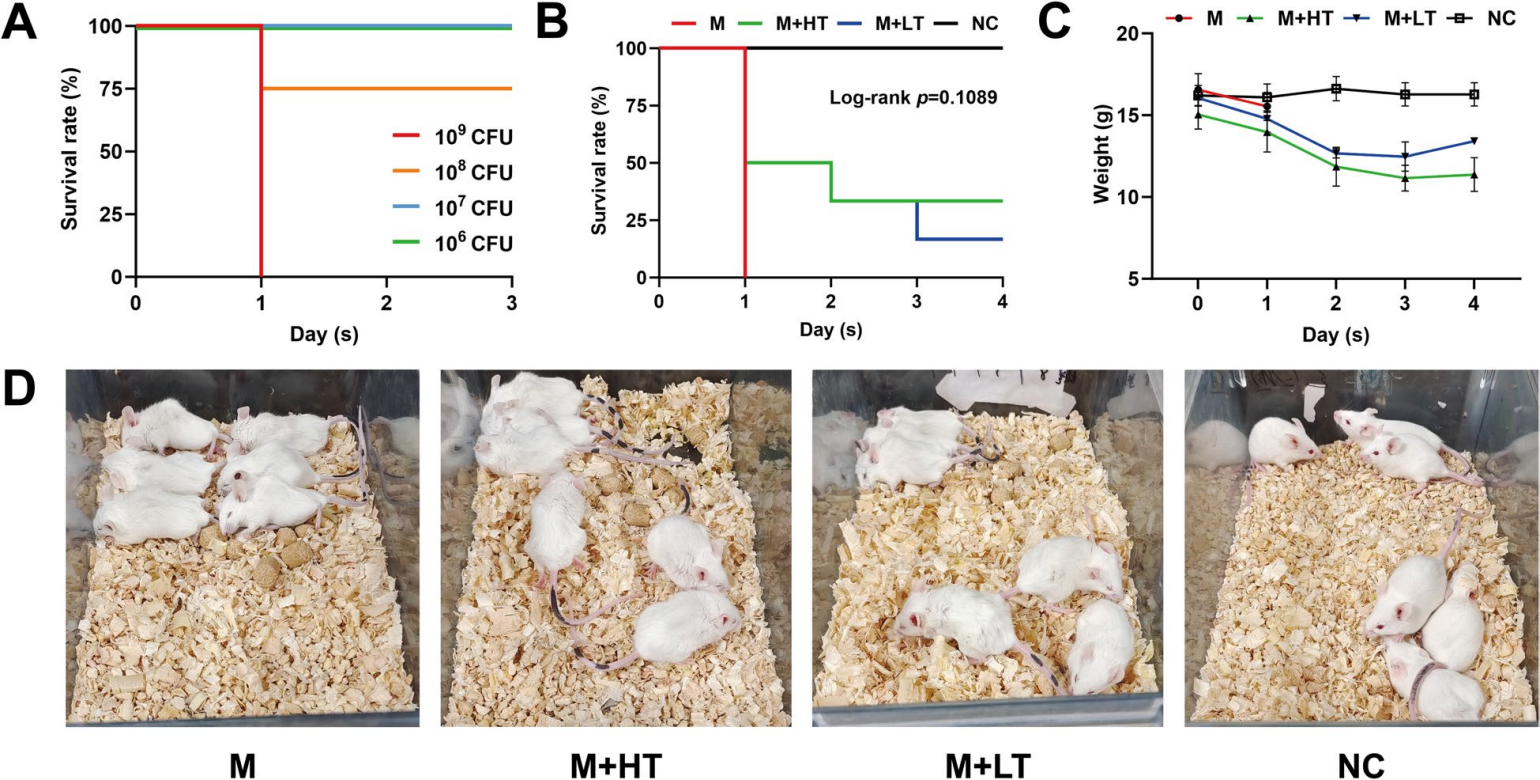
**Therapeutic efficacy of phage vB_EcoStr-FJ63A in bacteremic mice**. **A** Establishment of the bacteremic mouse model (*n* = 4). **B** Survival rates of mice after phage therapy (*n* = 6). **C** Changes in body weight of mice after phage therapy. **D** Clinical manifestations in mice after 1 day of phage therapy. Survival data were analyzed using the Kaplan–Meier method, and statistical differences between groups were assessed with the log-rank test. The significance level was set at *p* < 0.05.

Mice injected intraperitoneally with *E. coli* RN24 developed clinical symptoms including lethargy, ruffled fur, and fecal soiling around the anus. As shown in Figure 6A, an intraperitoneal injection of 10^9^ CFU of *E. coli* RN24 resulted in 100% mortality (LD_100_ = 10^9^ CFU), while 10^8^ CFU caused one death (MLD = 10^8^ CFU).

In the bacteremia model established with the LD_100_ dose of *E. coli* RN24, untreated mice exhibited severe lethargy and died within 1 day. For mice treated with high-dose (5 × 10^8^ PFU) or low-dose (5 × 10^6^ PFU) phage vB_EcoStr-FJ63A, 50% survival was observed in both groups on day 1 (three deaths per group; Figure 6B). Surviving mice remained lethargic, displayed ruffled fur, and experienced weight loss (Figures 6C, D). On day 2, one additional death occurred in each treatment group. By day 3, one more death was recorded in the low-dose group. No further deaths occurred thereafter. By day 4, surviving mice in both treatment groups showed improved activity, smoother fur, and partial weight recovery. Kaplan–Meier survival analysis revealed that both the high-dose and low-dose phage treatment groups had no significantly higher survival rate compared with the untreated infection model group (log-rank test, *p* = 0.1089). These results suggest that intraperitoneal administration of phage vB_EcoStr-FJ63A confers partial protection against *E. coli* RN24-induced bacteremia in mice.

### Antimicrobial activity and titer dynamics of phage vB_EcoStr-FJ63A in mice blood

**Figure 7:**
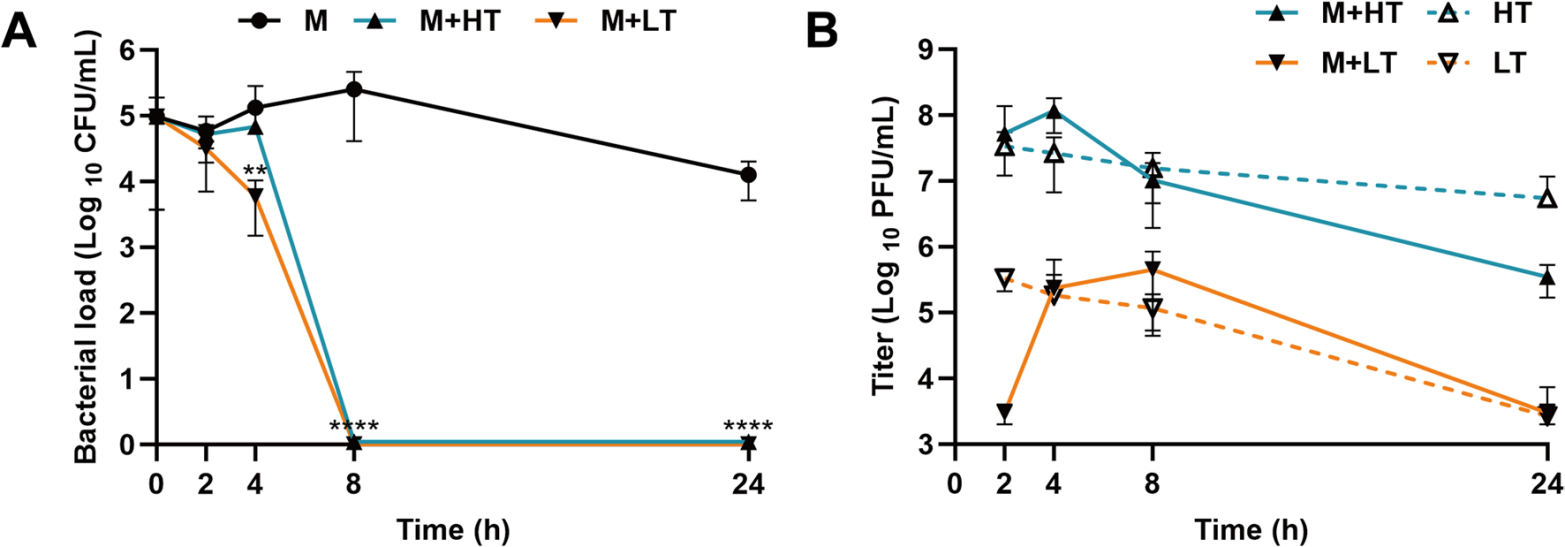
**Treatment of bacteremic mice with phage vB_EcoStr-FJ63A**. **A** Changes of bacterial load in bacteremia mouse blood after phage therapy (*n* = 3 per group at each time point). Statistical significance was determined by two-way ANOVA followed by Dunnett’s multiple comparisons test (versus model). Significance levels: **p < 0.01, ****p < 0.0001. **B** Phage kinetics in bacteremic versus healthy mice. Solid lines represent phage titers in mice infected with *E. coli* RN24 and subsequently treated with phage (bacteremic mice, *n* = 3 per group at each time point). Dashed lines represent the pharmacokinetic profile of phage administered to healthy, uninfected mice (historical data from the experiment described in “Pharmacokinetics of phage vB_EcoStr-FJ63A in mice blood” section and shown in Figure 5; *n* = 6 per group at each time point).

The antibacterial efficacy of phage vB_EcoStr-FJ63A in mice with bacteremia was further validated by measuring bacterial loads in the blood (Figure 7A). In the *E. coli* infection model group, mice injected intraperitoneally with the MLD of *E. coli* RN24 exhibited a bacterial concentration of 4.99 log _10_ CFU/mL in the blood at 2 h post-infection. The bacterial load continued to proliferate, reaching 5.96 log _10_ CFU/mL at 10 h post-infection (8 h post-phage treatment). By 26 h post-infection (24 h post-phage treatment), the *E. coli* RN24 concentration in the blood decreased to 4.10 log _10_ CFU/mL. In high-dose and low-dose treatment groups, the bacterial concentrations at 2 h post-phage administration showed no significant difference compared with the model group. However, at 4 h post-treatment, the bacterial loads in the high-dose and low-dose groups were reduced to 4.83 log _10_ CFU/mL and 3.12 log _10_ CFU/mL, respectively. Only the bacterial load in the low-dose group was significantly lower than than the model group’s 5.12 log _10_ CFU/mL (*p* = 0.0096). Remarkably, no *E. coli* RN24 was detected in the blood of either treatment group at 8 h and 24 h post-phage administration (The limit of detection was 3 log_10_ CFU/mL). Since the bacterial load in the untreated infection model group decreased 24 h post-infection, this suggests a contribution from the host immune system. Therefore, the above results indicate that phage therapy significantly accelerated the bacterial clearance process, leading to undetectable levels of bacteria in the blood within 8 h.

To compare the differences in the kinetics of phage titers between bacteremic mice and healthy mice, the pharmacokinetic profile of phage vB_EcoStr-FJ63A in the blood of healthy mice (data at the same time point after administration from the “Pharmacokinetics of phage vB_EcoStr-FJ63A in mice blood” section, groups receiving phage alone without bacterial challenge) was presented alongside the phage titers measured in bacteremic mice (Figure 7B). The changes in phage vB_EcoStr-FJ63A titers in the blood of high-dose and low-dose treatment groups, compared with its kinetics in healthy mice. Unlike in healthy mice, phage titers in the blood of bacteremic mice did not peak at 2 h post-injection. In the high-dose group, the titer continued to rise, reaching a maximum of 8.07 log _10_ PFU/mL at 4 h, while the low-dose group peaked at 8 h with 5.37 log _10_ PFU/mL. This suggests active phage replication through infection of *E. coli* RN24. By 24 h post-administration, phage titers declined in both treatment groups, mirroring the trend observed in healthy mice. However, the high-dose group exhibited a lower titer (~1.18 log _10_ PFU/mL) compared with healthy mice, this may be partly attributable to further activation of the host innate immune system by the bacterial infection. In contrast, the low-dose group’s phage titer closely resembled that of healthy mice. These findings highlight the dynamic interplay between phage replication, bacterial clearance, and host immunity in determining therapeutic outcomes.

## Discussion

In this study, we explored the molecular basis for the interaction between the phage vB_EcoStr-FJ63A and its host, and further evaluated its pharmacokinetics and therapeutic efficacy in a murine bacteremia model. Our findings provide insights for advancing this phage toward therapeutic applications.

Through phage adsorption assays, we observed that the adsorption capacity of phage vB_EcoStr-FJ63A to resistant mutants was significantly reduced, indicating that resistance in *E. coli* 63 primarily arises from impaired phage binding. Genomic analysis of the mutants revealed mutations in genes involved in the synthesis or regulation of OMPs, LPS, and extracellular polysaccharides [25–32]. These mutations likely alter or mask receptor structures, preventing phage adsorption and suggesting that vB_EcoStr-FJ63A may recognizes OMPs, LPS, and/or extracellular polysaccharides on the host surface.

To identify the receptor type for phage vB_EcoStr-FJ63A, we treated the surface polysaccharides and OMPs of the host strain *E. coli* 63 with sodium periodate and proteinase K, respectively, and assessed phage adsorption to the treated cells. The results demonstrated that both surface polysaccharides and OMPs influence phage adsorption, with polysaccharides playing a more critical role. This dual-receptor mechanism resembles that of phage T4, whose long tail fibers reversibly bind to OmpC and LPS on the surfaces of *E. coli* K12 and *E. coli* B, respectively, to initiate adsorption [33]. Given that mutations in resistant strains implicated LPS as a potential receptor for phage vB_EcoStr-FJ63A, we extracted LPS from *E. coli* 63 and the five resistant mutants and performed competitive inhibition assays. These experiments confirmed that LPS competitively inhibits phage adsorption, suggesting its role as a binding site. Studies on phage T4 reveal that its long and short tail fibers interact with LPS: the long fibers reversibly recognize the host, while the short fibers irreversibly anchor the phage to the surface [34]. Thomassen et al. showed that T4’s short tail fibers, composed of gp12 homotrimers, attach via their N-termini to the phage baseplate, with the C-termini serving as LPS-binding domains [35]. HHpred alignment revealed that the N- and C-termini of phage vB_EcoStr-FJ63A’s short tail fiber protein share 100% and 96.2% homology, respectively, with T4’s short tail fibers. This indicates that the short tail fibers of phage vB_EcoStr-FJ63A irreversibly bind LPS to anchor the phage to the host surface, explaining why polysaccharide oxidation by sodium periodate abolished adsorption by disrupting anchoring and subsequent DNA injection. However, LPS from resistant mutants also inhibited phage adsorption, implying that their LPS retains the receptor structure, and resistance likely arises from extracellular polysaccharides masking these sites.

To evaluate the role of OmpC in phage adsorption, we pretreated the surface polysaccharides of the host *E. coli* 63 with sodium periodate to minimize their interference. The experimental results showed that the number of phages adsorbed to the host surface after incubation with OmpC was lower than that observed with the truncated OmpC-Gln172*, but the difference was not statistically significant. This may be because of the inherently low adsorption efficiency of the phage in the absence of surface polysaccharides. To further explore the interaction between phage receptor-binding proteins (RBPs) and OmpC, we simulated protein–protein interactions. Predicting three-dimensional protein structures is a prerequisite for such simulations, and AlphaFold2 is one of the most widely used deep learning tools for this purpose [36]. AlphaFold3 extends this capability by predicting interactions between proteins, DNA, RNA, and ligands [37]. SWISS-MODEL template searches revealed homology between the long tail fiber adhesin of phage vB_EcoStr-FJ63A and gp38 of phage vB_SenM-S16. In gp38, the C-terminal domain mediates host specificity, while the N-terminal attaches to the gp37 trimer to form the long tail fiber [38]. Template searches for *E. coli* OmpC confirmed its typical homotrimeric structure [39]. AlphaFold3 predictions indicated that the C-terminal region of phage vB_EcoStr-FJ63A’s long tail fiber adhesin inserts into the cavity formed by the OmpC trimer. This aligns with findings by Islam et al., where RBPs at the distal end of long tail fibers interact with the OmpC cavity through hydrogen bonds, hydrophobic interactions, and van der Waals forces [40]. In addition, HHpred alignment demonstrated 100% homology probability (*E* = 4.8 × 10^−54^) between the long tail fiber adhesin of phage vB_EcoStr-FJ63A and gp38 of phage vB_SenM-S16. This structural and functional similarity is significant, as the long tail fibers of phage vB_SenM-S16 reversibly and specifically bind to *Salmonella* OmpC and LPS. These results from mutant analysis, competitive binding assays, and in silico modeling provide evidence for OmpC as a putative receptor for the long tail fiber adhesin of phage vB_EcoStr-FJ63A. However, its definitive validation as a functional receptor requires future genetic complementation studies to restore phage susceptibility in the relevant mutant strains.

To evaluate the pharmacokinetics and antibacterial effect of phage vB_EcoStr-FJ63A in mice, we first addressed key challenges in phage preparation, notably endotoxin removal. Endotoxins from Gram-negative bacteria can provoke severe immune reactions [41], and intraperitoneal administration must meet strict endotoxin limits [42]. While traditional methods such as PEG/NaCl precipitation and chloroform extraction only partially reduce endotoxins [41], and 1-octanol treatment alone proved insufficient for our high-titer lysates [13], we adopted an integrated approach. By sequentially using 1-octanol, cross flow ultrafiltration, and commercial endotoxin removal resin, endotoxins were effectively reduced to a low level, although some phage loss occurred owing to potential binding of LPS to the phage tail [43].

Intraperitoneal injection of phages has been shown to facilitate rapid absorption and systemic distribution [44]. For phage vB_EcoStr-FJ63A, it was detected in the blood within 30 min post-injection, continued to rise until reaching peak titers at 2 h, remained detectable until 24 h, and was completely cleared by 48 h. This pharmacokinetic profile aligns with phages Kp_Pokalde_002 and PEV20, which also reached maximum blood titers at 1 h and 4 h, respectively, followed by gradual decline and clearance by 48 h [6, 11]. In contrast, phages vB_KoxP_ZX8 and PA_LZ7 exhibited shorter blood circulation times, being fully eliminated within 6–8 h [45, 46]. While phage clearance kinetics in the blood appear to depend on dose and particle size, discrepancies still exist among similar phages.

In a mouse bacteremia model, established with a lethal dose (LD_100_) of *E. coli* RN24, intraperitoneally administered phage vB_EcoStr-FJ63A demonstrated partial protective effect. While all mice in the untreated control group died within 24 h, both high-dose (5 × 10^8^ PFU) and low-dose (5 × 10^6^ PFU) phage treatment groups showed 50% survival at 24 h. The observed efficacy was lower than some reported studies, which may relate to strain-specific sensitivity and the growth activity of *E. coli* RN24 in vivo [47]. Although phage vB_EcoStr-FJ63A formed plaques on LB agar plates inoculated with *E. coli* RN24, the plaque size and clarity differed from those on the host strain *E. coli* 63, and the efficiency of plating (EOP) was about 0.1 (Additional file 1). The variable efficacy of single-phage therapy against different isolates underscores the potential utility of phage cocktails or phage-antibiotic combinations. However, the interaction between phages and antibiotics can be antagonistic, influenced by factors such as timing, concentration, and antibiotic class—some of which may inhibit phage replication or adsorption [48–50]. Therefore, thorough in vitro and in vivo evaluations are essential before clinical application of such combinations.

In a mouse bacteremia model established with the MLD of *E. coli* RN24, phage vB_EcoStr-FJ63A exhibited accelerated bacterial clearance. No bacteria were detected in the blood of mice in either the high-dose or low-dose treatment groups at 8 h post-administration. It is important to note that the observed reduction in bacterial load in the untreated control group by 24 h post-infection indicates a contribution from the host immune system in combating the infection. Therefore, the therapeutic effect of vB_EcoStr-FJ63A is more accurately described as synergizing with and dramatically accelerating this natural clearance process, rather than acting as the sole mechanism of bacterial elimination. Interestingly, the antibacterial efficiency of the high-dose phage treatment did not surpass that of the low-dose group as anticipated, which may be attributed to individual variability and the limited sample size. This may indicate that MOI has an impact on the efficiency of phage therapy in clearing bacteria. In the high-dose group, the multiplicity of infection (MOI) in the blood exceeded 100, which implies that dozens or even hundreds of phage particles could simultaneously adsorb onto the surface of a single bacterial cell. This oversaturated attack may have led to a phenomenon analogous to the “prozone effect” in immunology, where excessively high antibody concentrations paradoxically inhibit antigen–antibody reactions, resulting in diminished detection signals. The phage baseplate hub structural protein of vB_EcoStr-FJ63A exhibits lysozyme R activity, which could cause premature and excessive disruption of the cell wall, potentially leading to its collapse before DNA injection is completed or the replication cycle is finalized [9]. In addition, the binding of a large number of tail fiber proteins to receptors may physically block porins or other channels, or even cause irreversible damage to the cell membrane, thereby interfering with normal physiological functions, including the injection of phage DNA. Moreover, the bacterial cells, in response to the massive phage invasion, may exhaust substantial resources and energy, rendering them unable to support the effective replication of any single phage. These factors collectively may explain why the bacterial lysis efficiency of the high-dose group was lower than that of the low-dose group. We also acknowledge that the small sample size (*n* = 3 per group at each time point) means that individual animal variation could influence the mean values at a single time point. The overall trend across the entire time course (i.e., no bacteria were detected in the blood of mice at 8 h and 24 h in both phage treatment groups) remains a relatively robust finding. This is an important area for future investigation, including more frequent sampling and mathematical modeling of phage–bacteria interactions in vivo.

Furthermore, the kinetics of phage titers in the blood of bacteremic mice differed markedly from those in healthy mice. Unlike healthy mice, where phage titers peaked at 2 h post-intraperitoneal injection, neither the high-dose nor low-dose treatment groups in the bacteremia model exhibited this trend. Instead, both groups displayed self-replicating properties, with titers peaking at 4 h (high-dose) and 8 h (low-dose), consistent with findings by Dhungana et al. [6]. This self-replicating capability creates a dynamic bidirectional interaction between phages and bacteria. Following infection and replication, progeny phages are released to initiate new infection cycles, thereby amplifying phage titers and enhancing bactericidal efficiency. In addition, the observed reduction in phage titer in the high-dose treatment group of bacteremic mice, compared with healthy mice, suggests complex in vivo dynamics. This accelerated clearance in bacteremic mice is likely due, in part, to the activation of the host innate immune system in response to the bacterial infection, which is known to enhance the removal of viral particles from the bloodstream [44]. Nonetheless, further studies specifically measuring immune markers and phage biodistribution are needed to confirm this mechanism. This dynamic interplay complicates the PK and PD of phage therapy, underscoring the need for expanded PK/PD studies in infected animal models to optimize therapeutic strategies.

Lastly, we reiterate that although this work provides insights into the receptor usage and in vivo efficacy of phage vB_EcoStr-FJ63A, it is subject to several limitations. First, the identification of OmpC as a putative receptor, while supported by multiple lines of evidence, lacks the definitive validation that genetic complementation experiments would provide. Second, our pharmacokinetic analysis was confined to the bloodstream, and the distribution of vB_EcoStr-FJ63A to major organs and tissues remains unknown, leaving its full biodistribution and potential sites of replication unclear. Third, although our in vivo efficacy study demonstrated bacterial clearance, the limited sample size led to a discrepancy from the expected results. Finally, the inclusion of additional in vivo controls, such as a group treated with heat-inactivated phage, would have helped to further delineate the specific contribution of active phage replication versus innate immune stimulation by phage virions or residual endotoxins. Addressing these points in future work will be critical for advancing the therapeutic development of vB_EcoStr-FJ63A.

## Conclusions

This study suggests that LPS and OMPs serve as binding sites for phage vB_EcoStr-FJ63A. We hypothesize that the long tail fiber adhesin specifically recognizes and reversibly binds to OmpC and LPS on the surface of the host *E. coli* 63, while its short tail fibers irreversibly anchor to LPS. In addition, we indicate that intraperitoneal administration of phage vB_EcoStr-FJ63A enables its entry into the murine blood, where it exhibits accelerated clearance and confers partial protection against bacteremia caused by colistin-resistant *E. coli*.

## Supplementary Information

**Additional file 1. Information of the E. coli strains used in this study.**

Additional file 2. Nucleic acid sequences of ompC and ompC-Gln172*.

**Additional file 3. Amino acid sequences of phage long tail fiber adhesin and OmpC.**

**Additional file 4. Virulence factors of E. coli 63 and RN24.**

Additional file 5. Expression and purification of OmpC and OmpC-Gln172*.

## Data Availability

Data will be made available on request.
