# Supplementary material for: Receptor identification and in vivo efficacy of a lytic phage vB_EcoStr-FJ63A against colistin-resistant Escherichia coli
Source: Vet Res. 2026 Jan 3;57:23. doi: 10.1186/s13567-025-01687-6 (PMC12857141; doi:10.1186/s13567-025-01687-6)
Supplement: Supplementary file 1 — Additional file 1. Information of the E. coli strains used in this study. [file 13567_2025_1687_MOESM1_ESM.docx]

**Additional file 1.** Information of the *E. coli* strains used in this study.

| Strains | EOP | Area | Source |
| --- | --- | --- | --- |
| 63 | 1 | Shandong | Chicken |
| 63M100R1 | 0 |  | Resistant mutant strain |
| 63M100R3 | 0 |  | Resistant mutant strain |
| 63M1R1 | 0 |  | Resistant mutant strain |
| 63M001R4 | 0 |  | Resistant mutant strain |
| 63M001R8 | 0 |  | Resistant mutant strain |
| RN24 | 0.1 | Guangxi | Dog |
| BL21(DE3) |  | Beijing | Tsingke Biotechnology Co., Ltd |
