# Supplementary material for: Receptor identification and in vivo efficacy of a lytic phage vB_EcoStr-FJ63A against colistin-resistant Escherichia coli
Source: Vet Res. 2026 Jan 3;57:23. doi: 10.1186/s13567-025-01687-6 (PMC12857141; doi:10.1186/s13567-025-01687-6)
Supplement: Supplementary file 2 — Additional file 2. Nucleic acid sequences of ompC and ompC-Gln172*. [file 13567_2025_1687_MOESM2_ESM.docx]

**Additional file 2.** Nucleic acid sequences of *ompC* and *ompC*-Gln172*.

| *ompC* | atgaaagttaaagtactgtccctcctggtcccagctctgctggtagcaggcgcagcaaacgctgctgaagtttacaacaaagacggcaacaaattagatctgtacggtaaagtagacggcctgcactatttctctgacaacaagtcagaagacggcgaccagacctatgtacgtcttggtttcaaaggcgaaactcaggttactgaccagctgaccggttacggccagtgggaatatcagatccagggcaatacctctgaagacaacaaagaaaactcctggacccgtgtggcattcgcaggtctgaaattccaggatgtaggttctttcgactacggtcgtaactacggcgttgtttacgatgtaacttcctggaccgacgtactgccagaattcggtggcgacacctacggttctgacaacttcatgcagcagcgtggtaacggcttcgcgacctaccgtaacaccgacttcttcggtctggttgacggtctgaactttgctgttcagtaccagggcaaaaacggtagcgtaagcggcgaaggcatgaccaacaatggtcgtggtgctctgcgtcagaatggcgacggtgtcggcggatctatcacttatgattacgaaggcttcggtatcggtgctgcagtttccagctccaaacgtactgatgatcaaaatggtagctacaccagcaatggtgtagttcgtaactacatcggtactggcgaccgtgctgaaacctacactggtggtctgaaatacgacgctaacaacatctacctggctgctcagtacacccagacctacaacgcaactcgcgtaggttccctgggttgggcgaacaaagcacagaacttcgaagctgttgctcagtaccagttcgactttggtctgcgtccgtccctggcttacctgcagtctaaaggtaaaaacctgggtgtcatcaatggtcgtaactacgacgacgaagatatcctgaaatatgttgatgttggtgcgacctactacttcaacaaaaacatgtccacctacgttgactacaaaatcaacctgctggacgacaaccagttcactcgtgacgctggcatcaacactgataacatcgtagctctgggtctggtttaccagttctaa |
| --- | --- |
| *ompC*-Gln172* | atgaaagttaaagtactgtccctcctggtcccagctctgctggtagcaggcgcagcaaacgctgctgaagtttacaacaaagacggcaacaaattagatctgtacggtaaagtagacggcctgcactatttctctgacaacaagtcagaagacggcgaccagacctatgtacgtcttggtttcaaaggcgaaactcaggttactgaccagctgaccggttacggccagtgggaatatcagatccagggcaatacctctgaagacaacaaagaaaactcctggacccgtgtggcattcgcaggtctgaaattccaggatgtaggttctttcgactacggtcgtaactacggcgttgtttacgatgtaacttcctggaccgacgtactgccagaattcggtggcgacacctacggttctgacaacttcatgcagcagcgtggtaacggcttcgcgacctaccgtaacaccgacttcttcggtctggttgacggtctgaactttgctgttcagtactagggcaaaaacggtagcgtaagcggcgaaggcatgaccaacaatggtcgtggtgctctgcgtcagaatggcgacggtgtcggcggatctatcacttatgattacgaaggcttcggtatcggtgctgcagtttccagctccaaacgtactgatgatcaaaatggtagctacaccagcaatggtgtagttcgtaactacatcggtactggcgaccgtgctgaaacctacactggtggtctgaaatacgacgctaacaacatctacctggctgctcagtacacccagacctacaacgcaactcgcgtaggttccctgggttgggcgaacaaagcacagaacttcgaagctgttgctcagtaccagttcgactttggtctgcgtccgtccctggcttacctgcagtctaaaggtaaaaacctgggtgtcatcaatggtcgtaactacgacgacgaagatatcctgaaatatgttgatgttggtgcgacctactacttcaacaaaaacatgtccacctacgttgactacaaaatcaacctgctggacgacaaccagttcactcgtgacgctggcatcaacactgataacatcgtagctctgggtctggtttaccagttctaa |
