# Supplementary material for: Receptor identification and in vivo efficacy of a lytic phage vB_EcoStr-FJ63A against colistin-resistant Escherichia coli
Source: Vet Res. 2026 Jan 3;57:23. doi: 10.1186/s13567-025-01687-6 (PMC12857141; doi:10.1186/s13567-025-01687-6)
Supplement: Supplementary file 3 — Additional file 3. Amino acid sequences of phage long tail fiber adhesin and OmpC. [file 13567_2025_1687_MOESM3_ESM.docx]

**Additional file 3.** Amino acid sequences of phage long tail fiber adhesin and OmpC.

| Phage long tail fiber adhesin | MAISSGWVGSSAVSETGQRWMSAAMEAVRLGRPAYMSAMVGRSKEIHYSIGANHNYNKDTLINYLKSQGSTPVVVTITGDLVSNSAGVPCLDFPSSLTNEYVTLIINPGVTVYGRGGRGAQAGNRAGQAGGTAINNGIGTRLRITNNGAIAGGGGGGGAQSTDNSWAGKYVSGGGGGRPFGAGGSNGAKYPGGAASLTSPGAGGKQWVGITWYGGDGGNVGERGKDAARANGFSNSPGAAGKAVTGNAPRWDKVGTIYGARV |
| --- | --- |
| OmpC | MKVKVLSLLVPALLVAGAANAAEVYNKDGNKLDLYGKVDGLHYFSDNKSEDGDQTYVRLGFKGETQVTDQLTGYGQWEYQIQGNTSEDNKENSWTRVAFAGLKFQDVGSFDYGRNYGVVYDVTSWTDVLPEFGGDTYGSDNFMQQRGNGFATYRNTDFFGLVDGLNFAVQYQGKNGSVSGEGMTNNGRGALRQNGDGVGGSITYDYEGFGIGAAVSSSKRTDDQNGSYTSNGVVRNYIGTGDRAETYTGGLKYDANNIYLAAQYTQTYNATRVGSLGWANKAQNFEAVAQYQFDFGLRPSLAYLQSKGKNLGVINGRNYDDEDILKYVDVGATYYFNKNMSTYVDYKINLLDDNQFTRDAGINTDNIVALGLVYQF |
