# Supplementary material for: Receptor identification and in vivo efficacy of a lytic phage vB_EcoStr-FJ63A against colistin-resistant Escherichia coli
Source: Vet Res. 2026 Jan 3;57:23. doi: 10.1186/s13567-025-01687-6 (PMC12857141; doi:10.1186/s13567-025-01687-6)
Supplement: Supplementary file 4 — Additional file 4. Virulence factors of E. coli 63 and RN24. [file 13567_2025_1687_MOESM4_ESM.docx]

**Additional file 4.** Virulence factors of *E. coli* 63 and RN24.

| *E. coli* 63 | *E. coli* RN24 |
| --- | --- |
| curlin major subunit CsgA | Enteroaggregative immunoglobulin repeat protein |
| intimin-like adhesin FdeC | AraC negative regulator |
| Type 1 fimbriae | Heat-stable enterotoxin EAST-1 |
| Avian *E. coli* haemolysin | Outer membrane hemin receptor |
| lipoprotein NlpI precursor | Colicin ia |
| Tellurium ion resistance protein | curlin major subunit CsgA |
| Tia Invasion determinant | *Salmonella* HilA homolog |
|  | Putative type I secretion outer membrane protein |
|  | intimin-like adhesin FdeC |
|  | Type 1 fimbriae |
|  | Glutamate decarboxylase |
|  | hemolysin expression modulator Hha (previous rmoA) |
|  | Avian *E. coli* haemolysin |
|  | Hemolysin F |
|  | Heat-resistant agglutinin |
|  | Adherence protein |
|  | Aerobactin synthetase |
|  | Ferric aerobactin receptor |
|  | lipoprotein NlpI precursor |
|  | Outer membrane protease (protein protease 7) |
|  | Outer membrane usher P fimbriae |
|  | homologs of the *Shigella flexneri* SHI-2 pathogenicity island gene shiA |
|  | Iron transport protein |
|  | Tellurium ion resistance protein |
|  | Protein TraJ (Positive regulator of conjugal transfer operon) |
|  | Outer membrane protein complement resistance |
|  | Outer membrane lipoprotein, YHD fimbriael cluster |
|  | Usher, YHD fimbriael cluster |
|  | Chaperone, YHD fimbriael cluster |
|  | Major pilin subunit, YHD fimbriael cluster |
