# Supplementary material for: Receptor identification and in vivo efficacy of a lytic phage vB_EcoStr-FJ63A against colistin-resistant Escherichia coli
Source: Vet Res. 2026 Jan 3;57:23. doi: 10.1186/s13567-025-01687-6 (PMC12857141; doi:10.1186/s13567-025-01687-6)
Supplement: Supplementary file 5 — Additional file 5. Expression and purification of OmpC and OmpC-Gln172*. [file 13567_2025_1687_MOESM5_ESM.docx]

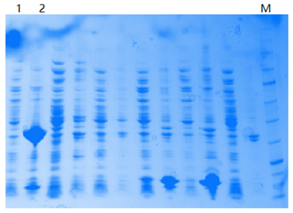


Fig. 1. Expression of OmpC. M: Marker; 1: Supernatant after cell lysis; 2: Precipitate after cell lysis.


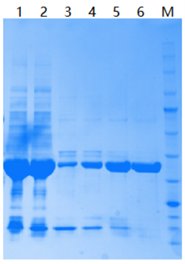


Fig. 2. Purification of OmpC. M: Marker; 1: Protein sample before loading; 2: Flow-through; 3: 25 mM imidazole **elution buffer**; 4: 50 mM imidazole **elution buffer**; 5: 100 mM imidazole **elution buffer**; 6: 200 mM imidazole **elution buffer.**


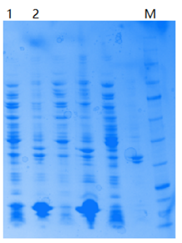


Fig. 3. Expression of OmpC-Gln172*. M: Marker; 1: Supernatant after cell lysis; 2: Precipitate after cell lysis.


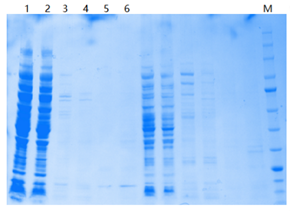


Fig. 4. Purification of OmpC-Gln172*. M: Marker; 1: Protein sample before loading; 2: Flow-through; 3: 25 mM imidazole **elution buffer**; 4: 50 mM imidazole **elution buffer**; 5: 100 mM imidazole **elution buffer**; 6: 200 mM imidazole **elution buffer.**
